# Legume–grass mixtures with arbuscular mycorrhizal fungi improve plant physiology, optimizing carbon and nitrogen dynamics and forage quality in perennial living mulches

**DOI:** 10.3389/fpls.2026.1853351

**Published:** 2026-06-19

**Authors:** Neda Nikpour Rashidabad, Ashley D. Keiser, Masoud Hashemi

**Affiliations:** Stockbridge School of Agriculture, University of Massachusetts Amherst, Amherst, MA, United States

**Keywords:** carbon and nitrogen allocation, forage quality, legume–grass species mixtures, living mulch, root architecture

## Abstract

**Introduction:**

Perennial cover crops (also referred to as living mulches) represent an innovative management strategy that provides continuous soil cover throughout the year and supports organic production systems, agroforestry practices, and the adoption of sustainable agricultural systems. Although the benefits of annual cover crops are well documented, the potential benefit of perennial cover crops to enhance ecosystem services, including the potential for providing high-quality forage, remains underexplored.

**Methods:**

To better understand the potential benefits of perennial cover crop mixtures, we quantified changes in plant physiology, biomass, and measures of forage quality across single- and mixed-species of clover (*Trifolium ambiguum*) and Kentucky bluegrass (*Poa pratensis*). We applied varying ratios of clover and grass (C_100_, C_75_-G_25_, C_50_-G_50_, C_25_-G_75_, G_100_), with and without arbuscular mycorrhizal fungi (AMF) inoculation.

**Results:**

Clover dominant mixtures, particularly C_75_–G_25_, exhibited higher leaf chlorophyll content and produced the greatest shoot biomass. This mixture, when inoculated with AMF, also showed the highest shoot C and N concentrations, with AMF increasing shoot and root C by 7–9% and N by 20–33%. Across monocultures and mixtures, AMF inoculation increased photosynthetic rates by 7.2% and reduced intercellular CO_2_ concentrations by 4.1%, which was associated with greater C and N concentration. AMF inoculation also enhanced root network depth, width, surface area, and volume by 3.5–28.1% across all treatments. Forage quality was specific to the species, with clover providing higher protein, fat, and calcium contents, and grass providing greater dry matter, phosphorus, potassium, lignin, ADF, NDF, and dNDF_48_.

**Discussion:**

Overall, our results demonstrate that mixed legume–grass systems, particularly when combined with AMF, optimized biomass production, C and N concentration, and forage quality.

## 1 Introduction

Perennial cover crops, also referred to as living mulches, provide continuous soil cover (Nikpour-Rashidabad et al., 2025) and maintain persistent living roots (Tang et al., 2022), delivering year-round ecosystem benefits compared to conventional annual cover crops (Schlautman et al., 2021). As perennial ground covers, they can serve additional functions, including forage production (Quinby, 2022; Quintarelli et al., 2022). Combining perennial grasses and legumes in mixed stands has the potential to enhance these benefits and increase their multifunctionality. Legumes supply atmospheric nitrogen (N), which can be transferred to companion grasses, thereby improving nutrient status and photosynthetic performance (Thilakarathna et al., 2016; Luo et al., 2024). In a mixed forage, legumes contribute protein while grasses provide carbohydrates; however, species mixtures can alter both the quantity and composition of harvestable biomass compared with monocultures (Sleugh et al., 2000; Tahir et al., 2022).

Interspecific competition for light and nutrients may strongly influence plant physiological performance (Eskelinen et al., 2022). For example, shading of white clover leaves by elongating petioles or neighboring grasses can reduce photosynthetic capacity by up to 30%, thereby altering carbon (C) assimilation, biomass allocation, and overall growth (Dennis and Woledge, 1983; Braun et al., 2010). Potential belowground interactions, including altered root length, depth, and lateral spread, can further shape resource acquisition under competitive conditions (Homulle et al., 2022). Together, these above- and belowground interactions can influence both yield and nutritional value of mixed- species perennial cover crop systems.

Plant-soil symbioses, particularly associations with arbuscular mycorrhizal fungi (AMF), play a central role in improving plant physiology and productivity (Zhu et al., 2023; Wang et al., 2025). By forming extensive hyphal networks that connect neighboring plants, AMF facilitate nutrient exchange and root-to-root signaling (Tadrosova et al., 2025). These symbionts also promote modifications in root architecture, such as increased surface area, branching, and fine root proliferation, thereby enhancing soil resource and water acquisition (Basyal and Emery, 2021; Fan et al., 2025). Improved nutrient uptake supports chlorophyll biosynthesis and photosynthetic capacity, leading to higher chlorophyll content and CO_2_ assimilation in inoculated plants (Ye et al., 2022; Fan et al., 2024). As strong belowground C sinks, AMF can utilize up to ~20% of plant-fixed C for fungal growth (Bonfante and Desirò, 2014; Kaiser et al., 2015; Salmeron-Santiago et al., 2021). In doing so, they may indirectly stimulate photosynthesis by preventing feedback inhibition caused by C concentration in leaves (Gavito et al., 2019; Salmeron-Santiago et al., 2021). Since AMF can improve plant nutrient acquisition, they can also modify plant biomass partitioning (Shah et al., 2025) and improve tissue nutritional quality and digestibility, traits particularly important in forage systems (Salmeron-Santiago et al., 2021). In agroecological systems designed for dual purpose, agroecological services, and forage production, AMF-mediated benefits may enhance both forage biomass quantity and quality (Baslam et al., 2014), increasing protein concentration in legumes (Pereira et al., 2020) and carbohydrate accumulation in grasses (Li et al., 2019), while supporting overall biomass production (Chippano et al., 2020; Husein et al., 2022).

Although plant species mixtures and AMF associations independently contribute to agroecological intensification, their combined impacts on plant physiology, root architecture, C and N allocation, and forage quality remain underexplored. Elucidating these interactions is essential for optimizing legume–grass perennial cover crop systems that supply high-quality forage, particularly when biomass is harvestable in early spring (before a cash crop establishment) and autumn (after cash crop harvest).

This study aimed to assess how varying mixture ratios of AberLasting clover (Trifolium ambiguum) and Kentucky bluegrass (Poa pratensis), with and without AMF inoculation, influence plant physiology, root development, C and N partitioning, and forage quality compared to species monoculture. The study was conducted within the context of perennial living mulch systems developed for diversified agricultural production, particularly those seeking to maintain continuous vegetative cover while improving soil function and system resilience. The outcomes of this research may be particularly relevant for temperate cropping systems adopting regenerative and low-input management practices aimed at reducing soil degradation, enhancing belowground ecological processes, and integrating supplemental forage production into sustainable agricultural landscapes. We hypothesized (H_1_) that legume–grass mixtures would enhance canopy photosynthetic performance and promote a more extensive root system development with increased belowground C allocation. We further hypothesized (H_2_) that AMF inoculation would amplify plant physiological responses by improving chlorophyll content, photosynthetic efficiency, and biomass accumulation across both single- and mixed-species stands. As an integrated outcome of these processes, we hypothesized (H_3_) that mixed stands inoculated with AMF would enhance forage yield and improve nutritional quality compared with monocultures.

## 2 Materials and methods

### 2.1 Experimental design

We conducted a greenhouse experiment at the University of Massachusetts Amherst (Amherst, MA, USA) to assess the influence of AMF on plant growth, root exudation, soil microbial biomass and activity, and N dynamics in monoculture and mixed-species perennial cover crops. The study followed a two-factor design. The first factor included five seeding ratios of Aberlasting clover (*Trifolium ambiguum* M.B.) and Kentucky bluegrass (*Poa pratensis* L.): 100% clover (C_100_), 75% clover + 25% grass (C_75_-G_25_), 50% clover + 50% grass (C_50_-G_50_), 25% clover + 75% grass (C_25_-G_75_), and 100% grass (G_100_). Each seeding treatment was further divided into AMF-inoculated and non-inoculated groups, resulting in ten treatment combinations, arranged in a randomized complete block design with three replicates (N = 30).

Greenhouse conditions were maintained at approximately 24°C during the day and 20 °C at night, with a mean relative humidity of 39% and a light intensity of approximately 140 W m^-^² under a 13-hour photoperiod. Soil was collected in summer 2024 from the upper 20 cm of a field adjacent to the research greenhouse, sieved to 2 mm, and homogenized using an electric mixer. A subsample was submitted to the UMass Soil and Plant Tissue Testing Laboratory (Amherst, MA, USA) for baseline physicochemical analysis (**Table 1**). To eliminate indigenous mycorrhizal propagules, the soil was autoclave-sterilized twice at 120 °C for 30 minutes. Plastic pots (20 cm × 20 cm) were filled with 3.5 kg of sterilized soil. Saprotrophic microbial communities were reintroduced to the soil by adding 100 mL of microbial inoculum to each pot. The inoculum was prepared by mixing 50–100 g of fresh soil with 1.5 L of deionized water, shaking for 2 h, allowing the soil to settle for 1 h, and filtering the supernatant through a 53-µm sieve followed by Whatman No. 1 filter paper (11 µm). Additionally, 1 g of a rhizobial inoculant containing *Sinorhizobium meliloti* and *Rhizobium leguminosarum* biovar trifolii was applied to ensure effective nodulations of clover.

**Table 1:** Physicochemical properties of the experimental soil.

pH	OC (mg kg^-1^)	Total N (mg kg^-1^)	P (mg kg^-1^)	K (mg kg^-1^)	Mg (mg kg^-1^)	Ca (mg kg^-1^)	CEC (meq/100g)
6.3	36	0.09	12.7	53	151	769	10

For AMF treatments, 4.08 g of MycoApply^®^ Ultrafine Endo (Mycorrhizal Applications, USA) was incorporated into the soil along with the saprotrophic inoculant. According to the manufacturer, the AMF inoculant contains approximately 130,000 propagules per pound, including four AMF species: *Rhizophagus intraradices*, *Funneliformis mosseae*, *Claroideoglomus etunicatum*, and *Claroideoglomus claroideum*. These species are known to associate with clover (Xie et al., 2021; Medina, 2022; Meng et al., 2022) and grasses (Liu et al., 2022; Aracely et al., 2025). Soils were incubated for 14 days before seeding to allow microbial stabilization.

Seeding rates were determined based on recommended field rates, pot surface area, and target species proportions: C_100_ = 0.042 g clover; C_75_-G_25_ = 0.0315 g clover + 0.02437 g grass; C_50_-G_50_ = 0.021 g clover + 0.0488 g grass; C_25_-G_75_ = 0.0105 g clover + 0.0732 g grass, and G_100_ = 0.0976 g grass. Seeds were evenly distributed over the soil surface and lightly covered on June 19, 2024. Pots were irrigated to field capacity and maintained without fertilizer application for 120 days to avoid interference with biological nitrogen fixation and to assess clover-derived nitrogen contribution and transfer under the experimental conditions.

### 2.2 Plant measurements and harvest

#### 2.2.1 Chlorophyll a, b, and total chlorophyll

One week before harvest, leaf chlorophyll a, b, and total chlorophyll were measured following Arnon (1949). Briefly, fresh leaf tissue (0.2 g) was extracted overnight in 80% acetone at − 4 °C, centrifuged at 10,000 x *g* for 5 min, and the absorbance of the supernatant was read at 645 and 663 using a spectrophotometer (Shimadzu UV-160A).

#### 2.2.2 Gas exchange measurements

One week before harvest, net photosynthesis rate, intercellular CO_2_ concentration, stomatal conductance, transpiration rate, and incident photosynthetically active radiation (PARi) were measured on fully expanded, healthy leaves (upper canopy position) of clover and grass using a portable photosynthesis system (LI-COR 6400, LI-COR Inc., Lincoln, NE, USA), with measurements conducted between 10:00 and 12:00 h on two consecutive days under ambient CO_2_ conditions.

#### 2.2.3 Harvest

At harvest, two representative plants were sampled from each mixed-species pot, one clover and one grass individual, and one plant from each monoculture pot for root architectural analysis. Intact root systems were carefully removed, gently rinsed, and scanned immediately (Section 2.5). The remaining plant material was separated into shoots and roots. Biomass per pot was determined after oven-drying at 75 °C to constant weight.

##### 2.2.3.1 Forage quality

Dried samples were ground using a Foss Cyclotec 1093 mill (Foss, Hilleroed, Denmark) fitted with a 0.42 mm screen. Subsamples were analyzed by near-infrared reflectance spectroscopy (NIRS) to determine crude protein, acid detergent fiber (ADF), digestible neutral detergent fiber at 48 h (dNDF_48_), lignin, ash, fat, and mineral concentrations (Ca, P, K, Mg). The NIRS instrument was calibrated against standard wet chemistry procedures to ensure analytical accuracy.

### 2.3 C and N concentrations, and C: N Ratio

Dried shoot and root samples were finely ground using a SPEX mixer mill (Cole Parmer, NJ, USA). Total C and N concentrations were determined at the University of Kansas Soil Analyses Service Center (Lawrence, KS, USA) using a Thermo FlashSmart Elemental Analyzer (Thermo Fisher Scientific). Carbon -to-nitrogen (C: N) ratios were calculated on a mass basis.

### 2.4 Root architectural trait analysis

Root system architecture was evaluated using GiA Roots software, a high-throughput image analysis tool for root trait quantification (Galkovskyi et al., 2012). Digital root images were captured using a Sony Cyber-Shot DSC-W510 camera (12.1 MP) and processed to obtain root architectural traits, including network length (total root length, mm), network depth (maximum vertical extent of the root system, mm), network width (maximum horizontal spread, mm), network surface area (total projected root surface area, mm²), and network volume (estimated three-dimensional root volume, mm³).

### 2.5 Statistical analysis

The experiment included two main factors: species mixture and AMF inoculation (presence or absence). Plant species was considered as an additional factor only for analyses of photosynthetic pigments and gas exchange parameters. Data were analyzed using a two-way factorial arrangement in a randomized complete block design with three replicates, using Minitab 22 (version 22.1). Means were separated using Tukey’s Honest Significant Difference (HSD) test, at *p* ≤ 0.05.

## 3 Results

### 3.1 Photosynthetic pigments

**Figure 1:**
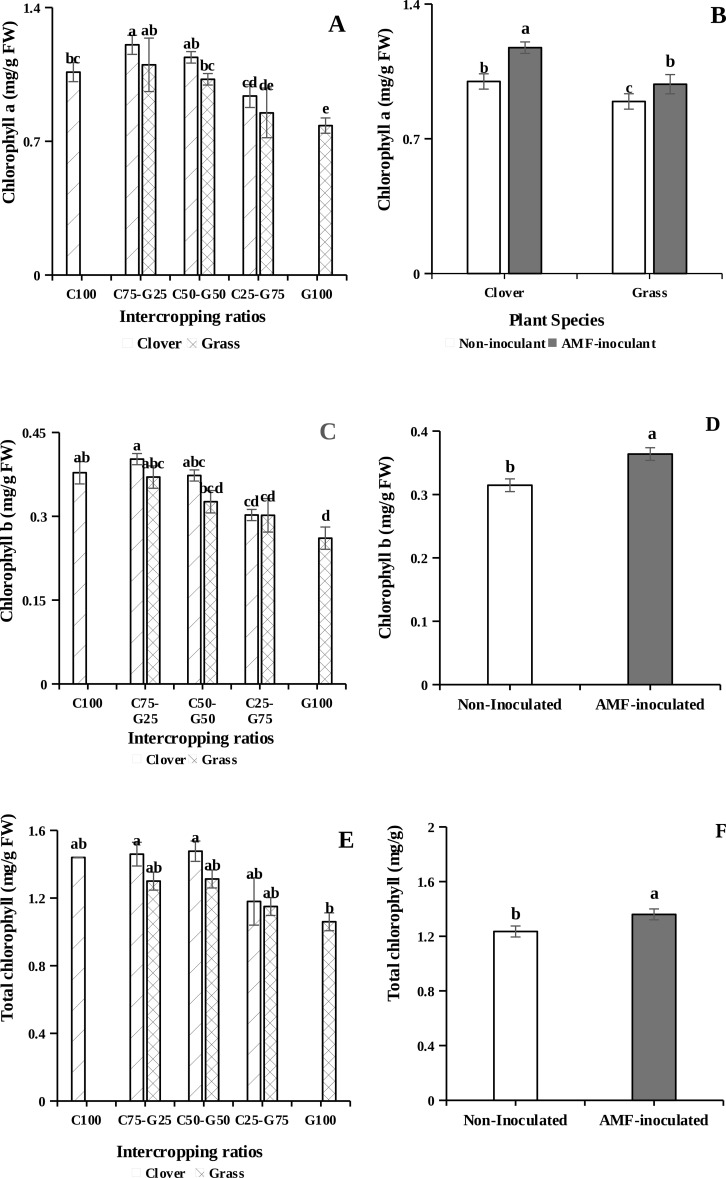
The impacts of monoculture and different species mixture ratios **(A, C, E)** and AMF inoculation **(B, D, F)** on chlorophyll a, b, and total of Aberlasting clover and Kentucky bluegrass, respectively. C_100_ = 100% clover; C_75_-G_25_ = 75% clover+ 25% grass; C_50_-G_50_ = 50% clover+50% grass; C_25_-G_75_ = 25% clover+ 75% grass; G_100_ = 100% grass. Data represent the average ± standard error (n=3). Letters indicate significant differences (p ≤ 0.05).

Across both species, chlorophyll a was significantly influenced by interactions between species × species mixtures (p ≤ 0.01) and species × AMF inoculation (p ≤ 0.01). Differences in chlorophyll b and total chlorophyll were influenced by a species × species mixtures interaction and AMF inoculation (p ≤ 0.01 and p ≤ 0.05, respectively). Chlorophyll a and b contents in clover peaked in the C_75_–G_25_ mixture, whereas increasing the grass proportion to 75% resulted in marked reductions (**Figure 1**). In contrast, grass benefited from greater clover presence in the mixture, where chlorophyll a and b reached maximum levels when grass accounted for 25% of the mixture. Total chlorophyll followed a similar pattern, with clover showing consistently higher values than grass in monoculture and mixtures, although these differences were not statistically significant (**Figures 1A, C, E**). AMF inoculation significantly enhanced chlorophyll a by 18.2% in clover and 9.2% in grass, while chlorophyll b and total chlorophyll increased by 16.1% and 9.7%, respectively (**Figures 1B, D, F**).

### 3.2 Photosynthetic parameters

**Figure 2:**
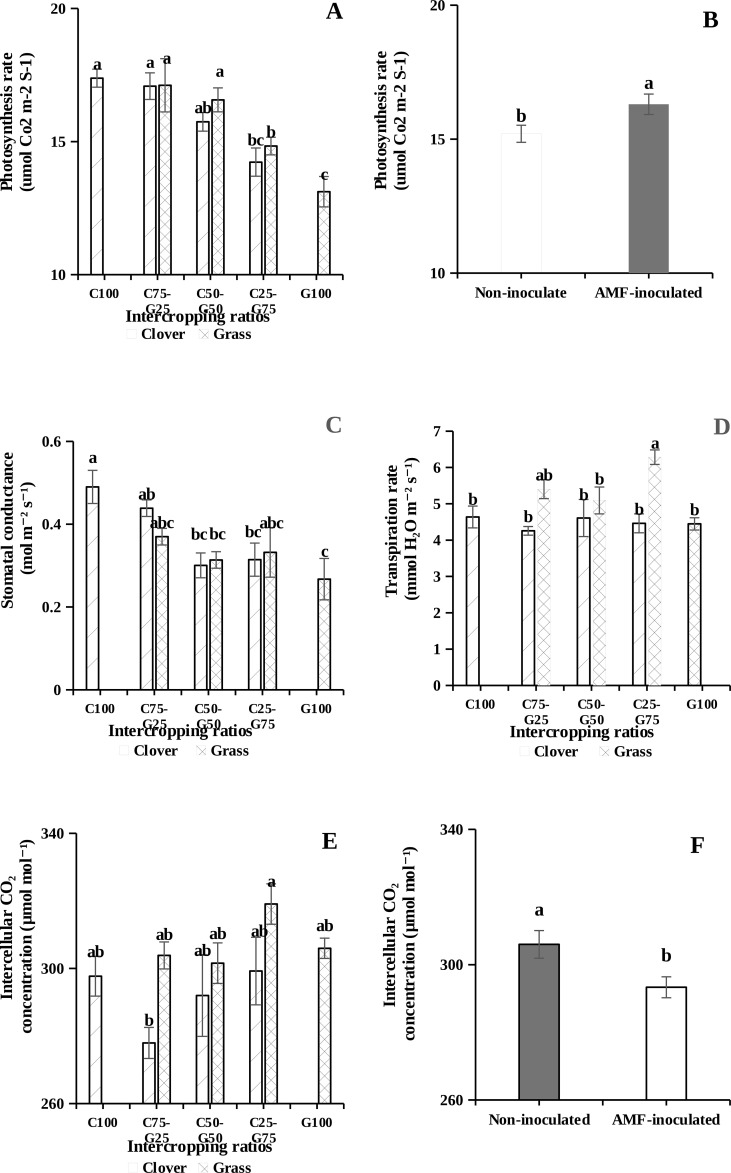
Effects of monoculture and different species mixture ratios on CO₂ uptake rate, total stomatal conductance, transpiration rate, and intercellular CO₂ concentration of clover and grass **(A, C, D, E)**, and effects of AMF inoculation on CO_2_ uptake rate and intercellular CO_2_ concentration **(B, F)**. C100 = 100% clover; C75-G25 = 75% clover+ 25% grass; C50-G50 = 50% clover+50% grass; C25-G75 = 25% clover+ 75%, grass; G100 = 100% grass. Data represent the average ± standard error (n=3). Letters indicate significant differences (p ≤ 0.05).

Species mixtures significantly affected photosynthetic rate (p ≤ 0.01), intercellular CO_2_ concentration (p ≤ 0.05), stomatal conductance (p ≤ 0.05), and transpiration rate (p ≤ 0.01). Overall, clover exhibited a 4.54% higher photosynthetic rate than grass. The presence of clover in the mixtures with clover increased photosynthetic rate and stomatal conductance in grass, particularly in clover-dominant stands. Conversely, increasing the proportion of grass reduced these parameters in clover. Clover achieved its highest photosynthetic rate and stomatal conductance in monoculture and in the C_75_–G_25_ mixture, whereas grass reached peak values when it accounted for 25% of the mixture (**Figures 2A, C**). Transpiration rate and intercellular CO_2_ concentration in clover were not significantly altered by species mixtures, whereas grass exhibited a significant increase in transpiration rate under species mixtures, particularly in the C_25_–G_75_ mixed ratio. The lowest intercellular CO_2_ concentration in clover was observed in the C_75_–G_25_ mixture (**Figure 2E**). Photosynthetic rate and intercellular CO_2_ concentration were also significantly influenced by AMF inoculation (p ≤ 0.01). AMF increased photosynthetic rate by 7.23% while reducing intercellular CO_2_ concentration by 4.14% (**Figure 2B**). No significant interaction was detected between species mixtures and AMF inoculation (**Figure 2**).

### 3.3 Shoot, root, and root/shoot ratio

Species mixtures and AMF inoculation significantly affected shoot and root biomass (p ≤ 0.01). Root-to-shoot ratio (R:S) was only affected by species mixtures (p ≤ 0.01). A significant interaction between species mixtures and AMF inoculation was detected only for shoot biomass (p ≤ 0.05). Shoot biomass was consistently greater in mixtures than in monocultures. The AMF-inoculated C_75_-G_25_ mixture produced the highest aboveground biomass (25.9 g), followed by the AMF-inoculated C_50_-G_50_ (23.7 g) and C_25_-G_75_ (22.7 g) treatments, which did not differ significantly from each other but exceeded both clover and grass monocultures. The lowest shoot biomass was recorded in monocultures, particularly in the non-inoculated grass monoculture (12.8 g) (**Table 2**). Root biomass and R:S ratio were highest in the grass monoculture (3.4) and lowest in the clover monoculture (1.3). Increasing the proportion of grass in mixtures resulted in greater root biomass and higher R:S ratios. Although AMF inoculation significantly enhanced both shoot and root biomasses, it had no significant impact on the R:S ratio (**Table 2**).

**Table 2:** Significance test of variation sources (mean squares) and their effects on shoot and root biomass, and Root/Shoot ratio.

Treatments		Shoot biomass (g)	Root biomass (g)	Root: shoot ratio
Replication		0.02 ns	10.7 ns	0.03 ns
Species mixture ratios (IR)		168.1 **	656.8 **	4.9 **
AMF-inoculation (AMF)		14.1 **	175.6 **	0.1 ns
IR × AMF		1.6 *	5.6 ns	0.01 ns
IR				
C_100_		14.3 ± 0.3 c	19.4 ± 0.8 d	1.3 ± 0.06 c
C_75_-G_25_		24.3 ± 0.7 a	30.8 ± 1.1c	1.3 ± 0.008 c
C_50_-G_50_		23.3 ± 0.3 ab	36.5 ± 0.9 bc	1.5 ± 0.04 bc
C_25_-G_75_		23.3 ± 0.3b	42.3 ± 0.8 ab	1.9 ± 0.02 b
G_100_		13.2 ± 0.2 c	45.9 ± 0.7a	3.4 ± 0.03 a
AMF				
Non-inoculated		18.8 ± 1.2 b	32.6 ± 1.6 b	1.8 ± 0.02
Inoculate		20.2 ± 1.3 a	37.4 ± 1.6 a	1.9 ± 0.03
IR × AMF				
Non-inoculated	C_100_	13.8 ± 0.3 cd	18.3 ± 1.3	1.3 ± 0.06
	C_75_-G_25_	22.7 ± 0.5 b	27.7 ± 0.9	1.2 ± 0.01
	C_50_-G_50_	22.9 ± 0.4 b	33.1 ± 1.5	1.4 ± 0.08
	C_25_-G_75_	21.9 ± 0.5 b	39.7 ± 0.5	1.8 ± 0.02
	G_100_	12.8 ± 0.2 d	44 ± 05	3.5 ± 0.03
AMF-inoculated	C_100_	14.9 ± 0.2 c	20.4 ± 0.9	1.4 ± 0.06
	C_75_-G_25_	25.9 ± 0.2 a	33.8 ± 1.4	1.3 ± 0.005
	C_50_-G_50_	23.7 ± 0.3 b	39.9 ± 1.0	1.7 ± 0.008
	C_25_-G_75_	22.7 ± 0.3 b	44.9 ± 1.2	2.0 ± 0.02
	G_100_	13.7 ± 0.2 cd	47.8 ± 1.0	3.5 ± 0.04

Data represents the average ± standard error.

S, Species; AMF, AMF-inoculation; C, Clover; G, Grass.

** and *, significant at 1% and 5% probability levels, respectively.

Different letters between the treatments indicate significant differences at p ≤ 0.05.

### 3.4 C and N contents, C:N ratio, and C and N yields of shoots and roots

Mixtures of clover and grass significantly affected C and N contents, C:N ratio, and C and N yields in both shoots and roots (p ≤ 0.01). Across all treatments, C and N concentrations were consistently greater in shoots than in roots.

Grass-dominant treatments (G_100_, C_25_-G_75_, and C_50_-G_50_) exhibited higher shoot C content, whereas clover-dominant treatments (C_100_ and C_75_-G_25_) showed greater root C content. Clover monoculture (C_100_), followed by C_75_-G_25_ mixture, produced the highest N concentrations in both shoots and roots. Increasing the grass proportion to ≥ 50% resulted in a significant decline in N content. Consequently, the C:N ratio in both shoots and roots was highest in the grass monoculture (G_100_) and decreased progressively with increasing clover proportion, reaching the lowest values in C_100_. Species mixtures also significantly influenced N yield. The highest shoot N yield (N concentration x biomass) was calculated in the C_75_-G_25_ mixture, whereas mixtures containing >50% clover produced the highest root N yields (**Table 3**).

**Table 3:** Significance test of variation sources (mean squares) and their effects on TC, TN, TC/TN, C, and N yields of Aberlasting clover and Kentucky Bluegrass shoot and roots.

Treatments		C concentration (mg g^-1^)		N concentration (mg g^-1^)		C:N ratio		C Yield (g)		N Yield (g)	
		Shoot	Root	Shoot	Root	Shoot	Root	Shoot	Root	Shoot	Root
Species mixture ratios (IR)		116.2 **	10126.6 **	89.8 **	216.5 **	39.4 **	463.0 **	28.2 **	15.7 **	0.2 **	0.04 **
AMF-inoculation (AMF)		169.9 **	2433.3 *	22.6 *	29.6 ns	4.3 ns	65.5 ns	3.1 **	25.3 **	0.04 **	0.1 **
IR × AMF		21.2 ns	230.7 ns	5.2 ns	4.7 ns	1.2 ns	24.5 ns	0.3 *	0.6 ns	0.001 **	0.009 ns
IR											
C_100_		402.4 ± 2.7 c	314.6 ± 8.9 a	31.6 ± 0.6a	20.1 ± 1.8 a	12.7 ± 0.2 c	16.2 ± 3.7 d	5.8 ± 0.1 c	6.1 ± 0.4 c	0.4 ± 0.001 c	0.4 ± 0.04 ab
C_75_-G_25_		407.8 ± 1.3 bc	280.5 ± 8.2 a	29.7 ± 1.4 ab	16.4 ± 1.5 a	13.9 ± 0.7 bc	17.6 ± 2.9cd	9.9 ± 0.3a	8.6 ± 0.5 b	0.7 ± 0.05 a	0.5 ± 0.06 a
C_50_-G_50_		409.5 ± 1.0 ab	230.5 ± 10.2 b	28.0 ± 0.9 ab	9.5 ± 1.2 b	14.7 ± 0.5 bc	25.5 ± 2.5bc	9.5 ± 0.2 ab	8.4 ± 0.8 b	0.6 ± 0.02 b	0.4 ± 0.06 ab
C_25_-G_75_		410.6 ± 0.7 ab	218.6 ± 6.5 b	26.5 ± 1.0 b	7.8 ± 0.8 b	15.6 ± 0.6 b	29.6 ± 3.0 ab	9.2 ± 0.1 b	9.2 ± 0.4 ab	0.6 ± 0.02 b	0.3 ± 0.04 b
G_100_		414.4 ± 2.4 a	229.9 ± 5.8 c	21.4 ± 0.8 c	6.2 ± 0.2 b	19.4 ± 0.7 a	37.5 ± 1.3 a	5.5 ± 0.1c	10.6 ± 0.5 a	0.3 ± 0.01 d	0.3 ± 0.02 b
AMF											
Non-inoculated		406.6 ± 1.4 b	245.8 ± 10.9 b	26.6 ± 1.0 b	13.0 ± 1.6	15.6 ± 0.7	26.7 ± 2.4	7.6 ± 0.5 b	7.7 ± 0.4 b	0.5 ± 0.04 b	0.3 ± 0.02 b
Inoculate		411.3 ± 1.3 a	263.8 ± 10.4 a	28.3 ± 1.1 a	11.0 ± 1.5	14.9 ± 0.7	23.8 ± 2.3	8.3 ± 0.5 a	9.5 ± 0.5 a	0.6 ± 0.05 a	0.4 ± 0.03 a
IR × AMF											
Non AMF-inoculated	C_100_	399.1 ± 4.8	305.4 ± 8.6	31.3 ± 1.2	19.2 ± 3.5	12.8 ± 0.5	16.9 ± 2.7	5.5 ± 0.06 cd	5.6 ± 0.5	0.4 ± 0.01 d	0.3 ± 0.07
	C_75_-G_25_	405.3 ± 1.2	280.0 ± 14.5	27.2 ± 1.8	16.0 ± 0.4	15.0 ± 1.0	17.5 ± 1.0	9.2 ± 0.2 b	7.8 ± 0.8	0.6 ± 0.04 b	0.4 ± 0.04
	C_50_-G_50_	408.5 ± 0.9	213.5 ± 2.3	27.1 ± 1.8	7.1 ± 0.4	15.2 ± 1.0	30.5 ± 2.1	9.4 ± 0.2 b	7.1 ± 0.8	0.6 ± 0.04 b	0.2 ± 0.02
	C_25_-G_75_	410.5 ± 1.1	212.4 ± 2.5	26.2 ± 2.0	7.1 ± 0.9	15.9 ± 1.3	31.0 ± 4.6	8.9 ± 0.2 b	8.4 ± 0.2	0.5 ± 0.03 bc	0.3 ± 0.04
	G_100_	409.5 ± 2.3	217.7 ± 4.0	21.1± 0.6	5.8 ± 0.2	19.4 ± 0.7	37.9 ± 2.1	5.2 ± 0.1 d	9.6 ± 0.4	0.3 ± 0.005 e	0.2 ± 0.01
AMF-inoculated	C_100_	405.6 ± 2.1	323.7 ± 15.1	32.0 ± 1.7	21.1 ± 1.5	12.7 ± 0.07	15.5 ± 1.7	6.0 ± 0.1 c	6.6 ± 0.6	0.5 ± 0.01d	0.4 ± 0.01
	C_75_-G_25_	410.4 ± 0.9	281.0 ± 11.1	32.2 ± 1.0	16.8 ± 3.4	12.8 ± 0.3	17.6 ± 2.5	10.6 ± 0.07 a	9.5 ± 0.3	0.8 ± 0.03 a	0.6 ± 0.11
	C_50_-G_50_	410.5 ± 1.9	247.4 ± 15.2	28.2 ± 0.2	12.1 ± 0.6	14.2 ± 0.2	20.5 ± 1.5	9.7 ± 0.1 b	9.9 ± 1.0	0.7 ± 0.01 b	0.5 ± 0.06
	C_25_-G_75_	410.8 ± 1.1	224.8 ± 12.8	26.8 ± 0.9	8.4 ± 1.4	15.4 ± 0.5	28.2 ± 4.7	9.3 ± 0.1 b	10.0 ± 0.2	0.6 ± 0.02 b	0.4 ± 0.06
	G_100_	419.3 ± 1.1	242.2 ± 1.03	21.8 ± 0.3	6.6 ± 0.3	19.5 ± 1.5	37.0 ± 1.8	5.7 ± 0.1 cd	11.6 ± 0.4	0.3 ± 0.02 e	0.3 ± 0.02

Data represents the average ± standard error.

S, Species; AMF, AMF-inoculation; C, Carbon; N, Nitrogen.

** and *, significant at 1% and 5% probability levels, respectively.

Different letters between the treatments indicate significant differences at p ≤ 0.05.

AMF inoculation significantly influenced shoot C content, shoot C yield, shoot N yield, and root C and N yields (p ≤ 0.01), and also affected root and shoot N concentrations (p ≤ 0.05). Overall, across treatments, AMF inoculation increased C concentration and enhanced C and N yields in both shoots and roots, while also elevating shoot N concentration in both species. Significant species mixture × AMF interactions were detected for shoot N content (p ≤ 0.01) and shoot C yield (p ≤ 0.05), with the strongest interaction effects observed in the C_75_–G_25_ and C_50_–G_50_ mixtures. The highest shoot C and N yields were observed in the AMF-inoculated C_75_–G_25_ mixture, whereas the lowest values occurred in the non-inoculated grass monoculture (G_100_). In contrast, under AMF inoculation, the highest root C yield was observed in grass-dominant treatments (G_100_ and C_25_–G_75_) (**Table 3**).

### 3.5 Root structure

**Figure 3:**
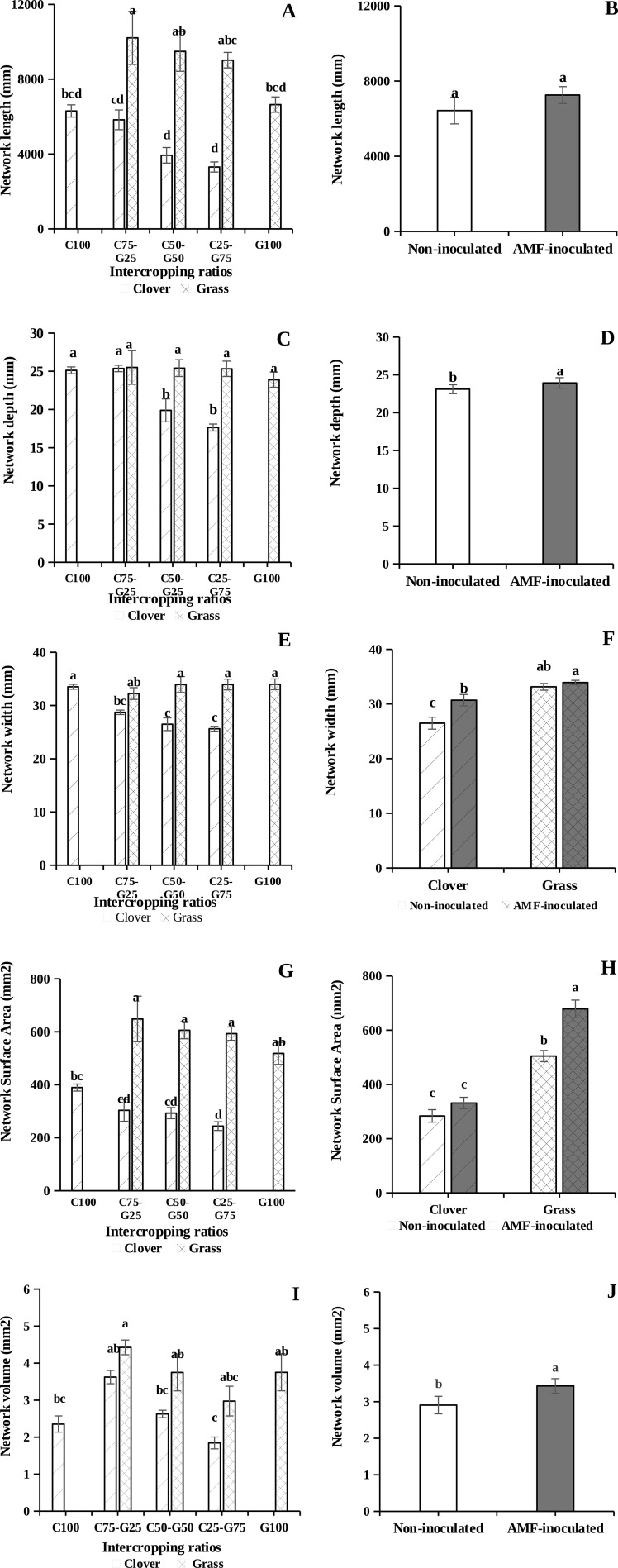
The impacts of monoculture and different species mixtures ratios **(A, C, E, G, I)** and AMF inoculation **(B, D, F, H, J)** on root network length (mm), depth (mm), width (mm), surface area (mm^2^), and volume (mm^2^) of Aberlasting clover and Kentucky bluegrass, respectively. C_100_ = 100% clover; C_75_-G_25_ = 75% clover+ 25% grass; C_50_-G_50_ = 50% clover+50% grass; C_25_-G_75_ = 25% clover+ 75% grass; G_100_ = 100% grass. Data represent the average ± standard error (n=3). Letters indicate significant differences (p ≤ 0.05).

In monoculture, clover and grass exhibited similar root network length, depth, width, and volume. However, species mixture proportions significantly affected root network width and depth (p ≤ 0.01). As the proportion of grass increased, clover showed progressive declines in root network length, depth, width, and volume, with the lowest values observed under grass-dominant conditions. (**Figures 3A, C, E, I**). In contrast, grass root systems remained relatively stable across mixture treatments, with no significant differences in root depth, root width, surface area and volume compared with the G_100_ monoculture (**Figures 3C, E, G, I**). Grass root length increased with increasing clover proportion and reached its maximum in the C_75_–G_25_ mixture (**Figure 3A**). Across all treatments, grass consistently exhibited greater root network surface area than clover, whereas clover surface area declined markedly as grass proportion increased (**Figure 3G**). Root network volume was greater in the grass monoculture than in the clover monoculture, peaked for both species in the C_75_–G_25_ mixture, and declined slightly when grass proportion exceeded 25% of the mixture. The lowest root network volume was recorded for clover in the C_25_–G_75_ mixture (**Figure 3I**).

In addition to species and mixture effects, AMF inoculation significantly enhanced root system development. AMF increased root network width and surface area (p ≤ 0.01), as well as root depth and volume (p ≤ 0.05). Across treatments, AMF inoculation increased root depth by 3.5%, width by 8.4%, surface area by 28.1%, and volume by 17.2% (**Figures 3D, F, H, J**). The increase in root width was more pronounced in clover (15.8%) than in grass (2.4%), whereas the enhancement of root surface area was greater in grass (34.5%) than in clover (16.8%) (**Figures 3F, H**).

### 3.6 Forage quality

Forage quality was higher in clover than in grass. Species mixtures of clover and bluegrass significantly affected crude protein, fat, calcium (Ca), phosphorus (P), potassium (K), lignin, acid detergent fiber (ADF), neutral detergent fiber (NDF), and 48-h *in vitro* digestible neutral detergent fiber (dNDF_48_) (p ≤ 0.01). Clover contained higher concentrations of crude protein (326.7 mg g^-1^), fat (3.72%), and Ca (10.6 mg g^-1^), whereas grass had higher P (4.1 mg g-1), K (25.5 mg g-1), lignin (4.98%), ADF (20.24%), NDF (43.04%), and dNDF_48_ (38.69%). The inclusion of bluegrass in mixtures reduced protein, fat, and Ca concentrations. However, crude protein levels did not differ significantly among C_100_, C_75_–G_25_, and C_50_–G_50_. Overall, mixture treatments exhibited intermediate values relative to the two monocultures. Notably, all mixtures containing grass showed statistically similar ADF concentrations and higher NDF and dNDF_48_ levels than the clover monoculture (C_100_), indicating that even low proportions of grass were sufficient to reduce clover digestibility (**Table 4**).

**Table 4:** Significance test of variation sources (mean square) and their effects on the forage quality of Aberlasting clover and Kentucky bluegrass.

Treatments	Protein (mg g^-1^)	Fat (%)	Ca (mg g^-1^)	P (mg g^-1^)	K (mg g^-1^)	Lignin (%)	ADF (%)	NDF (%)	dNDF_48_ (% of NDF)
Replication	122.8 ns	0.11 ns	0.4 ns	0.04 ns	4.0 ns	0.17 ns	1.35 ns	14.27 ns	4.49 ns
Species mixture ratios (IR)	965.4 **	3.01 **	39.9 **	0.3 **	12.7 **	11.91 **	24.56 **	132.86 **	262.041 **
AMF-inoculation (AMF)	197.4 ns	0.04 ns	0.9 ns	0.01 *	2.6 ns	2.37 *	12.04 **	13.32 ns	25.86 ns
AMF x IR	30.1 ns	0.05 ns	0.6 ns	0.001 ns	1.6 ns	0.34 ns	2.00 ns	1.38 ns	6.916 ns
IR									
C_100_	326.7 ± 7.2 a	3.72 ± 0.01 a	10.6 ± 0.06 a	3.6 ± 0.09 c	22.2 ± 0.6 b	1.18 ± 0.25 d	17.64 ± 0.76 b	30.83 ± 0.42 c	21.06 ± 0.21 d
C_75_-G_25_	326.3 ± 2.9 a	2.96 ± 0.20 b	8.6 ± 0.8 b	3.8 ± 0.1 bc	22.5 ± 0.5 b	2.31 ± 0.16 c	19.23 ± 0.59 ab	32.35 ± 1.30 bc	27.39 ± 2.26 c
C_50_-G_50_	307.9 ± 3.9 ab	2.70 ± 0.08 bc	7.7 ± 0.3 b	3.8 ± 0.05 bc	23.1 ± 0.4 ab	3.00 ± 0.14 bc	19.12 ± 0.60 ab	32.35 ± 0.80 bc	29.71± 0.34 bc
C_25_-G_75_	304.5 ± 2.6 b	2.25 ± 0.07 cd	5.8 ± 0.2 c	4.0 ± 0.04 ab	24.8 ± 0.9 ab	3.48 ± 0.46 b	19.96 ± 0.26 a	35.40 ± 1.71 b	33.49 ± 0.57 b
G_100_	299.6 ± 4.0 b	1.84 ± 0.12 d	3.9 ± 0.2 d	4.1± 0.04 a	25.5 ± 0.6 a	4.98 ± 0.09 a	20.24 ± 0.42 a	43.04 ± 0.38 a	38.69 ± 0.60 a
AMF									
Non-inoculated	310.4 ± 3.6	2.65 ± 0.19	7.2 ± 0.7	3.8 ± 0.07 b	23.3 ± 0.4	3.27 ± 0.32 a	19.87 ± 0.19 a	35. 99 ± 1.40	30.99 ± 1.83
AMF-Inoculated	315.6 ± 4.3	2.73 ± 0.18	7.5 ± 0.6	3.9 ± 0.06 a	23.9 ± 0.6	2.71 ± 0.40 b	18.87 ± 0.50 b	34.66 ± 1.15	29.15 ± 1.54

Data represents the average ± standard error.

S, Species; AMF, AMF-inoculation; C, Clover; G, Grass; Ca, Calcium; P, Phosphorus; K, Potassium; ADF, Acid Detergent Fiber; NDF, Neutral Detergent Fiber; dNDF_48_, 48-hour *in vitro* digestible neutral detergent fiber.

** and *, significant at 1% and 5% probability levels, respectively.

Different letters between the treatments indicate significant differences at p ≤ 0.0.5.

AMF inoculation significantly affected P and lignin concentrations (p ≤ 0.05) and ADF (p ≤ 0.01), while having limited effects on other forage quality parameters. Specifically, AMF inoculation increased P concentration by 2.6% and reduced lignin and ADF concentrations by 17.1% and 5.03%, respectively (**Table 4**). No significant species mixture × AMF interactions were detected for any forage quality parameters (**Table 4**).

## 4 Discussion

### 4.1 Influence of species mixtures and AMF on photosynthetic performance and biomass

Given the potential for cover crops to serve as dual-purpose systems, we explored how species mixtures versus monocultures impact forage biomass, physiological performance, and nutritional quality. Species mixtures are widely reported to enhance biomass production and overall soil health compared with monocultures due to complementary resource use and niche differentiation (Finney et al., 2016; Liu et al., 2025; Wendling et al., 2019). Consistent with this expectation and supporting H1, biomass accumulation was greater in mixed systems, particularly at the C_75_–G_25_ ratio (**Table 2**). Increasing plant diversity can improve overall N cycling (Schipanski and Drinkwater, 2012) and enhance plant N acquisition (Nyfeler et al., 2011). In our study, these benefits appear to be associated with enhanced photosynthetic performance when clover comprised ≥ 50% of the mixture. Under the C_75_–G_25_ and C_50_–G_50_ treatments, higher chlorophyll a and b concentrations in both species (**Figures 1A, C**) resulted in enhanced photosynthetic rates (**Figure 2A**), suggesting improved light harvesting and C assimilation. When the grass proportion exceeded 25% of the mixture, competitive dynamics shifted. Grass likely outcompeted clover, as exhibited by greater plant height and more extensive root development, including higher root biomass (**Table 2**), root length (**Figure 3A**), and root surface area (**Figure 3G**). These morphological traits confer advantages in light interception, water acquisition, and nutrient uptake (Li et al., 2021). Increase in stomatal conductance in grass under mixed conditions (**Figure 2C**), especially in the C_25_–G_75_ treatment, was accompanied by higher transpiration rates (**Figure 2D**) and intracellular CO_2_ concentrations (**Figure 2E**), reflecting altered gas exchange dynamics. In contrast, clover in the C_75_–G_25_ mixture exhibited the lowest intracellular CO_2_ concentration along with elevated clover chlorophyll content (**Figure 1E**), suggesting more efficient CO_2_ fixation and enhanced photosynthetic capacity (**Figure 2A**). Both factors likely contributed to greater clover shoot biomass (**Table 2**). These findings align with previous studies showing that legume–grass interactions can modify leaf gas exchange traits; for example, undersown red clover has been reported to alter stomatal conductance and intercellular CO_2_ concentration in companion crops (Orzech et al., 2024).

AMF inoculation further enhanced photosynthetic performance (**Figure 2B**), likely through improved water and nutrient acquisition. Inoculated plants exhibited higher N accumulation (**Table 3**) and increased P concentration (**Table 4**) (Wu et al., 2024), both of which are essential for chlorophyll synthesis and photosynthetic metabolism (Fathi, 2022; Cakmak and Engels, 2024). Co-inoculation with AMF and rhizobia can stimulate clover nodulation and biological N fixation (Xie et al., 2020), while AMF hyphae facilitate N acquisition and interspecific N transfer in mixed systems (Luo et al., 2023). Enhanced N availability supports chlorophyll biosynthesis, whereas P is critical for ATP production (Lal and Bhatla, 2023), phosphorylated intermediates (Wagner, 2024), Calvin cycle sugar phosphates (Odoom and Ofosu, 2024), and the structural integrity of NADP(H) (Lu et al., 2025). Adequate P availability thus underpins energy transfer, C fixation, chlorophyll formation, and photosystem function (Khan et al., 2023). Collectively, these mechanisms led to higher photosynthetic rates (**Figure 2B**) and lower intracellular CO_2_ concentrations (**Figure 2F**) in AMF-inoculated plants, ultimately translating into greater shoot and root biomass (**Table 2**), supporting our H_2_. Overall, these findings confirm that species complementarity and AMF symbiosis act synergistically to enhance photosynthetic efficiency (Wu et al., 2022; Slimani et al., 2024; Ma et al., 2025) and biomass accumulation (Qin et al., 2023; Zhu et al., 2023; Yang et al., 2025). These results underscore the importance of optimizing species composition and microbial associations to improve productivity and sustainability in perennial mixed-species systems.

### 4.2 Influence of species mixtures and AMF on C and N partitioning

Inter- and intraspecific interactions regulate metabolite allocation and the cycling of C and N within and among species mixtures (Andersen et al., 2023). As an N_2_-fixing legume, clover allocated a greater proportion of C to roots to support nodulation, with rhizobia acting as a strong C sink to meet the high energy demand of biological N_2_ fixation (Parvin et al., 2020). Consequently, clover-dominant treatments exhibited the highest shoot and root N concentrations, which progressively declined as grass proportion increased (**Table 3**).

Grass, in contrast, allocated more C to shoots to optimize light capture, partly because greater leaf area enhances radiation interception and aboveground productivity (Irving, 2015). Because clover invested more C in the belowground to support nodulation and N fixation, it maintained relatively lower C:N ratios in both shoots and roots compared with grasses. In contrast, lower tissue N concentrations in grass-dominant treatments led to higher C:N ratios (**Table 3**), reflecting greater investment in structural biomass and slower N turnover. This shift may slow residue decomposition and affect N cycling and availability within the broader agroecosystem (Finney et al., 2016; da Mota Neto et al., 2024).

AMF inoculation further increased total C accumulation in both shoots and roots, reflecting improved assimilate partitioning (**Table 2**). The AMF hyphal network functions as an additional C sink (He et al., 2023; Solaiman, 2024), stimulating photosynthetic activity and promoting additional C assimilation (Ran et al., 2021; Khan et al., 2022). Shoot N concentration also increased following AMF inoculation, likely due to enhanced N uptake and transfer via hyphal networks and, in legumes, improved nodulation and biological N_2_ fixation (Gough et al., 2021; Liu et al., 2021, Liu et al., 2023b). Consequently, C and N yields in both shoots and roots were higher in AMF-inoculated plants, indicating more efficient N assimilation and biomass production (Wu et al., 2024). Shoot C and N yields closely followed biomass responses, with AMF-inoculated C_75_–G_25_ mixtures showing the highest values, highlighting a synergistic interaction between species mixtures and mycorrhizal colonization (Wang et al., 2021). Root C yield paralleled root biomass patterns, being greatest in grass-dominant mixtures due to their greater root systems. In contrast, root N yield peaked in clover-dominant treatments, reflecting elevated tissue N concentrations associated with biological N_2_ fixation. Overall, these findings align with H_2_ and previous findings that AMF symbiosis improves nutrient status and productivity in mixed-species systems (Dobo, 2022; Zhou et al., 2024; Li et al., 2025).

### 4.3 Influence of species mixtures and AMF on root structure

Species mixtures also induced structural modifications in root system architecture (Wambsganss et al., 2021; Liu et al., 2023). Although clover and grass exhibited similar root traits in monoculture, distinct adjustments emerged under mixed conditions (**Figure 3**). When grass exceeded 25% of the mixture, clover root length, depth, width, and volume declined. This reduction likely reflects increased competitive pressure from grass because shading and nutrient competition reduced photosynthetic rates (**Figure 2A**), thereby constraining the C availability for belowground growth. In contrast, grass maintained root depth and width and exhibited greater root length and volume as clover proportion increased, likely benefiting from N transfer from clover. Across all mixtures, grass consistently exhibited a larger root surface area than clover, whereas clover root surface area declined progressively with increasing grass dominance. These results indicate that interspecific interactions in mixed stands differentially shape root architecture, with clover being more sensitive to competitive suppression belowground. This finding supports H_1_, demonstrating that legume–grass mixtures alter root architectural traits and alter root network development.

AMF inoculation further stimulated root system development, increasing root depth, width, surface area, and volume. These improvements likely result from AMF-induced stimulation of root branching and elongation, mediated through improved hormonal signaling and enhanced nutrient and water availability in the rhizosphere (Liu et al., 2024; Fan et al., 2025). Moreover, the extensive extraradical hyphal network expands the effective absorptive zone, enabling more efficient acquisition of relatively immobile nutrients, particularly P and micronutrients (Wang et al., 2022; Zhang et al., 2023). Consequently, AMF-colonized plants developed more extensive and functionally efficient root systems, supporting greater resource acquisition and overall productivity in intercropped systems (Xu and Liu, 2024). These results align with H_2_, highlighting the role of mycorrhizal symbiosis in strengthening belowground structural development and resource-use efficiency in mixed-species systems.

### 4.4 Nutritional value of mixed forage in a dual-purpose living mulch system

Beyond growth and root traits, and consistent with our third hypothesis (H_3_), species mixtures significantly influenced the nutritional quality of the mixed grass-clover forage. These effects reflect the integrated consequences of biomass allocation patterns and nutrient acquisition strategies. Clover exhibited higher protein, fat, and Ca concentrations, consistent with its symbiotic N_2_ fixation capacity (Kebede, 2021). Enhanced N acquisition supports the synthesis of N-rich metabolites such as amino acids and proteins (**Table 4**) (Ahmed et al., 2023). Grasses, in contrast, invested more C in structural components, as evidenced by higher lignin, acid detergent fiber (ADF), and neutral detergent fiber (NDF) concentrations. This structural investment reduced digestibility but improved tissue rigidity and persistence. Collectively, the mixed-species treatments demonstrate the nutritional benefits of integrating perennial cover crops into dual-purpose systems where they function both as living mulch and forage. AMF inoculation further increased forage yield and nutritional values. Improved P uptake under AMF symbiosis was associated with reduced lignin and ADF contents, likely mediated by enhanced water uptake and shifts in C allocation from structural and secondary compounds toward metabolically active, digestible tissues. Similar improvements in forage quality and digestibility under AMF symbiosis have been reported in various legume–grass systems (Baslam et al., 2014; Chang et al., 2022; García-Parisi et al., 2025).

## 5 Conclusion

Although the primary objective of incorporating perennial cover crops is to enhance overall soil health and support soil biological activity, these crops can also be harvested or grazed before planting and after harvesting a cash crop such as corn. This dual-purpose use improves the economic viability of the integrated cropping system. This study evaluated plant physiology, biomass production, and forage quality of a dual-purpose living mulch system in the presence and absence of mycorrhizal inoculation. The results demonstrated that clover-grass mixtures at specific proportions, particularly C_75_-G_25_ and C_50_-G_50_, effectively enhanced biomass production while maintaining acceptable nutritional value as forage, underscoring the potential of legume–grass mixtures as dual-purpose living mulch systems. Increased forage quality within clover-dominated mixtures was supported by the complementary physiological and morphological traits of the two species. Clover’s root C allocation supported symbiotic N_2_ fixation and grass’s greater root biomass and volume enhanced N uptake for supporting shoot growth, facilitated efficient resource partitioning, optimized photosynthetic performance, and sustained higher overall productivity. AMF inoculation amplified these benefits by improving root architecture, nutrient uptake, and C assimilation. Species mixtures also moderated nutrient composition, balancing the high protein and mineral content contributed by clover with the structural carbohydrates of grass. Although small grass proportions reduced overall forage digestibility, the measured reductions provide a roadmap for selecting species mixtures that complement the use of the forage. Collectively, these results highlight the potential of integrating legumes, grasses, and mycorrhizal symbioses to increase harvestable biomass and forage quality while also reinforcing the multifunctional role of their agroecological benefits.

## Data Availability

The raw data supporting the conclusions of this article will be made available by the authors, without undue reservation.
